# Dupilumab in a 9-week-old with Netherton Syndrome Leads to Deep Symptom Control

**DOI:** 10.1007/s10875-024-01837-z

**Published:** 2024-11-15

**Authors:** Yannik Vollmuth, Narjes Abdulhameed Alelq, Franziska Sattler, Susanne Schmidt, Fabian Hauck

**Affiliations:** 1 Department of Pediatrics, Dr von Hauner Children’s Hospital, Ludwig-Maximilians-University Munich, Lindwurmstraße 4 , Munich, European Union (EU) D-80337 Germany; 2 Department of Dermatology and Allergology, University Hospital, LMU Klinikum, European Union (EU), Munich, Germany; 3 Ministry of Health, Dammam, Saudi Arabia

**Keywords:** Netherton syndrome, Allergic inflammation, Dupilumab, Early infancy

## Abstract

**Purpose:**

Netherton syndrome (NS) is a rare inborn error of immunity (IEI) with an incidence of approximately 1:200,000 and the phenotypic triad of trichorrhexis invaginate (bamboo hair), congenital ichthyosiform erythroderma, and multiple atopic manifestations. Treatment options especially in infants are scarce and generally not licensed.

**Methods:**

Case report of a 9-week-old infant with NS treated with dupilumab off-label.

**Results:**

We report rapid and sustained resolution of allergic inflammation, deep symptom control including normalization of the skin microbiome, and catch-up somatic and psychomotor development without adverse drug reactions.

**Conclusion:**

Due to the high complication rate of NS, especially in the first years of life, we recommend treatment with dupilumab off-label immediately after the diagnosis has been established.

**Supplementary Information:**

The online version contains supplementary material available at 10.1007/s10875-024-01837-z

## To the Editor

Netherton syndrome (NS) is a rare inborn error of immunity (IEI) with an incidence of approximately 1:200,000 and the phenotypic triad of trichorrhexis invaginate (bamboo hair), congenital ichthyosiform erythroderma, and multiple atopic manifestations [1].

NS is caused by biallelic loss-of-function (LOF) variants in the serine peptidase inhibitor Kazal type 5 (*SPINK5*) gene [1]. *SPINK5* codes for the lympho-epithelial Kazal-type-related inhibitor (LEKTI) that is highly expressed in the granular layer of the epidermis and hair follicles where it inhibits the kallikrein-related serine proteases (KLKs). LEKTI deficiency results in increased proteolytic activity of KLKs and leads to premature desquamation of the epidermis, degradation of the hair follicles, and a pronounced defect of the skin and gastrointestinale barriers [2].

Importantly, in keratinocytes KLK5 leads to the activation of the protease-activated receptor 2 (PAR2) and the canonical nuclear factor kappa B (NF-κB) signaling pathway. This leads to the production of thymic stromal lymphopoietin (TSLP), the differentiation of naïve helper T cells (T_H_0) into T helper type 2 cells (T_H_2), and the secretion of interleukin-4 (IL-4), IL-5, and IL-13 mediating allergic inflammation [3]. In addition, the barrier defect leads to the invasion of pathogens, which induces a T helper type 17 cells (T_H_17) mediated immune response with the production of further pro-inflammatory cytokines such as IL-17 A and IL17F [2]. In the first years of life, NS-associated barrier defects lead to life-threatening bacterial infections and allergic inflammation mediates tantalizing itch and accumulation of multiple atopic manifestations. In sum, NS interferes with proper development of affected individuals and heavily impacts on their quality of life as well as that of their social networks [4].

Dupilumab is a human immunoglobulin subclass 4 (IgG_4_) monoclonal antibody that binds the interleukin-4 receptor alpha subunit (IL-4Rα) and inhibits the signaling of IL-4 and IL-13. As a result, T_H_2-mediated allergic inflammation can be dampened and eventually controlled. Dupilumab has been used since 2017 for the management of moderate to severe atopic dermatitis in adults and children onwards from 6 months of age [5].

We here report for the first time the off-label-use of dupilumab started in a 9-week-old infant with NS leading to to rapid and sustained resolution of allergic inflammation, deep symptom control including normalization of the skin microbiome, and catch-up somatic and psychomotor development without adverse drug reactions.

The female patient was born by caesarean section at 35 + 6 weeks of gestational age with a birth weight of 3,180 g. Despite external heat supply, she showed hypothermia (34.1 °C), and was transferred to a neonatal intensive care unit. Shortly afterwards, hypernatriemic dehydration appeared. The entire skin was erythematous and scaly and therefore congenital ichthyosis was suspected. A biopsy was taken which showed subacute eczema. Molecular diagnostics using a primary genodermatosis gene panel revealed a known pathogenic homozygous *SPINK5* splice site variant (NM_006846.4; c.1431-12G > A). In total, the diagnosis of NS was made.

**Fig. 1:**
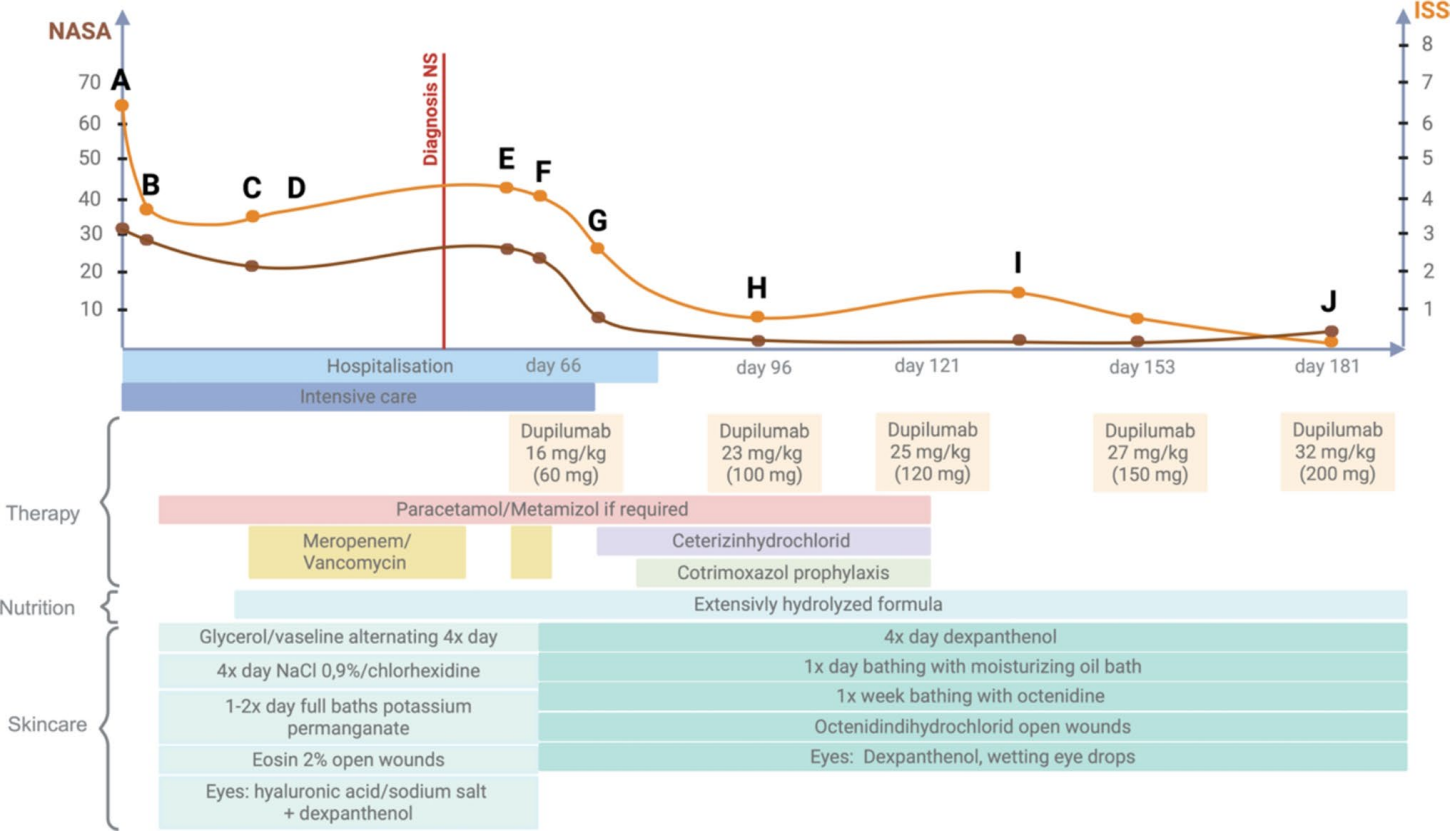
Correlation of dupilumab and external treatment with the skin phenotype of a toddler with Netherton syndrome

**Fig. 2:**
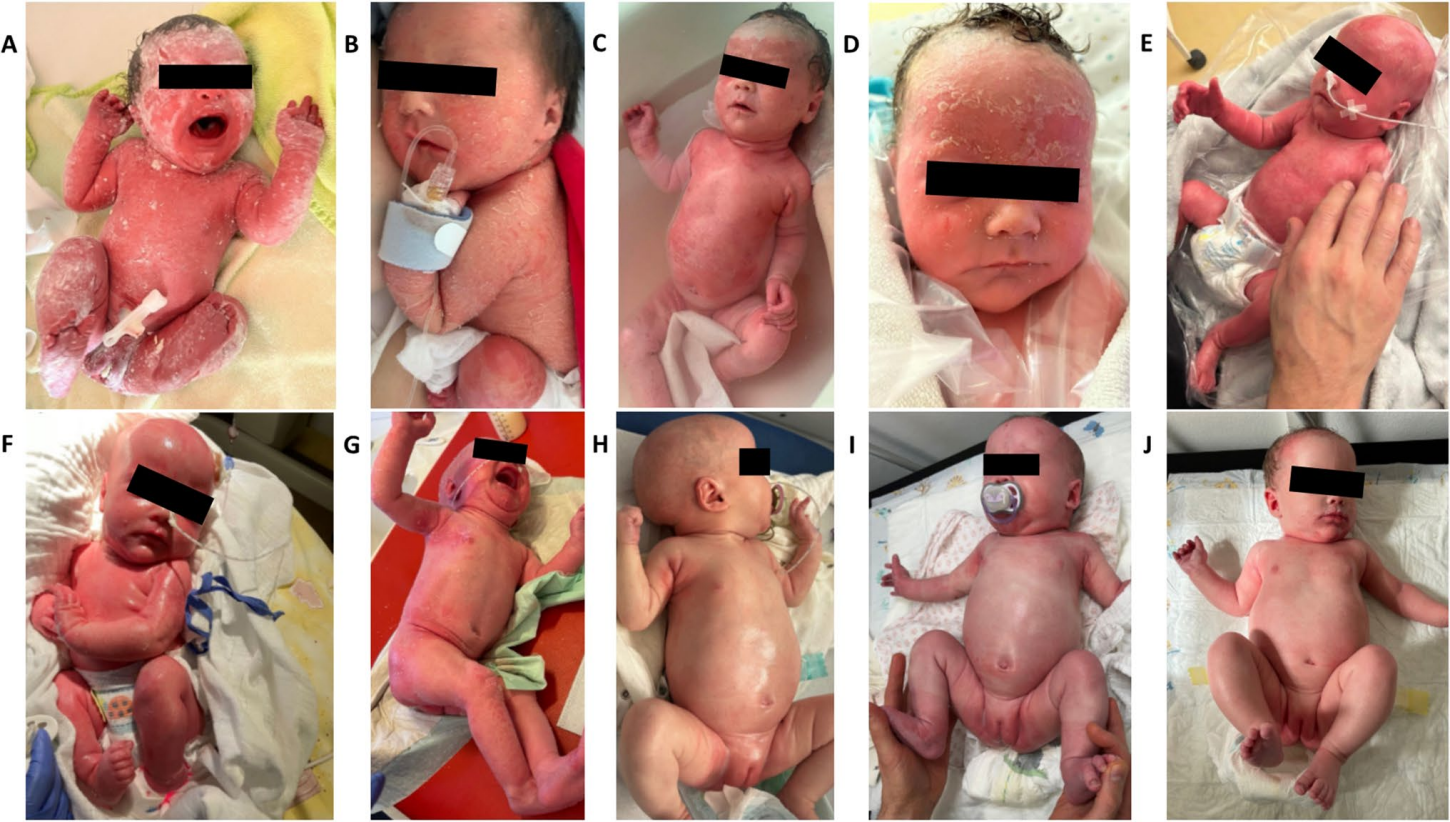
Photo documentation of the skin phenotype. **A** Erythroderma and scaling at birth, **B**-**D** variable degree of erythroderma and scaling before dupilumab tratment, **E**-**F** persisting of erythroderma despite scaling reduction, **G** obvious skin improvement seven days after the first dose of dupilumab, **H** one month after first dose, **I** hair regrowth after two doses, **J** four months after first dose. Written informed consent was obtained from the caregivers to publish the course of the therapy and the photo documentation

**Fig. 3:**
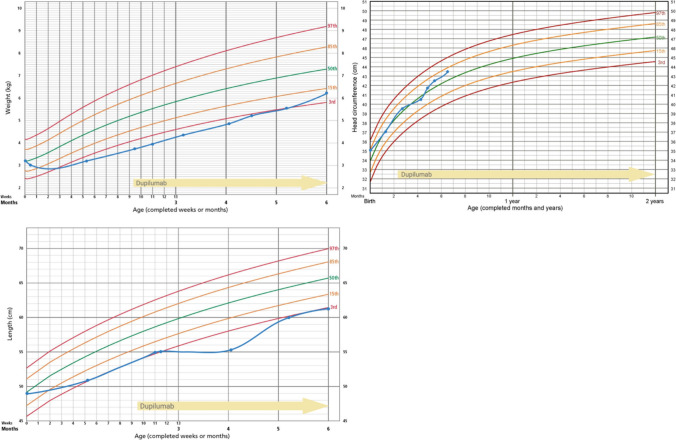
Growth based on the WHO percentiles

At the age of 8.5 weeks, the toddler was transferred to the Dr. von Hauner Children’s University Hospital because of treatment failure and multiple complications (see supplementary case report). We obtained written informed consent of the caregivers and absorption of costs by the public health insurance system and started dupilumab on day 66 of life with a dose of approximately 16 mg/kg (body weight 3.7 kg, 60 mg absolute dose). To asses treatment response, the Netherton Area Severity Assessment (NASA) and the Ichthyosis Scoring System (ISS) were applied [6, 7]. Figures 1 and 2 show the correlation between treatment and the skin phenotype over time. We noted a clear improvement of the skin condition already after the first week of dupilumab (Fig. 1 F-G). The patient could be discharged 14 days after the first dupilumab dose. Further doses were administered subcutaneously into the thigh and repeated approximately every 4 weeks in our immunodeficiency outpatient clinic. The dose was increased by 2–7 mg/kg each time until the target dose of approximately 32 mg/kg (body weight 6.2 kg, absolute dose 200 mg) was reached. After the second dose, the hair started growing again. Shortly before the third dose, all supportive medication was discontinued. The daily nutritional intake could be increased. The patient startetd sleeping through the night without further signs of itiching. Body weight increased from the 3rd to the 10th percentile, body heigth increased form below the 3rd percentile to the 3rd percentile (Fig. 3). Daily skin care was continued as described (supplementary case report and Fig. 1). The monthly dupilumab doses were continued at a dosage of 200 mg per month as a longterm treatment strategy. In the follow-up skin swabs at the age of 7.5 months, *Pseudomonas aeruginosa* and *MRSA* were only detected in the nose, while the entire body surface was parthogen-free. No side effects occurred with a follow-up of 20 weeks.

In summary, the combination of early-onset dupilumab and external treatment lead to rapid and sustained resolution of allergic inflammation and skin barrier defect resulting in deep symptom control and catch-up somatic and psychomotor development without adverse drug reactions in a toddler with NS. Due to the high complication rate of NS, especially in the first years of life, we recommend treatment with dupilumab immediately after the diagnosis has been established.

## Supplementary Information

Below is the link to the electronic supplementary material.

ESM 1 (DOCX. 12.4 KB)

## Data Availability

No datasets were generated or analysed during the current study.

## Abbreviations

IEI: Inborn error of immunity
IgG_4_: Immunoglobulin subclass 4
IL: Interleukin
IL-4Rα: Interleukin-4 receptor alpha subunit
ISS: Ichthyosis scoring system
KLK: kallikrein-related serine proteases
LEKTI: lympho-epithelial Kazal-type-related inhibitor
LOF: loss-of-function
NASA: Netherton area severity assessment
NF-κB: Nuclear factor kappa B
NS: Netherton syndrome
PAR2: Protease-activated receptor 2
SPINK5: Serine peptidase inhibitor Kazal type 5
T_H_0: Naïve helper T cell
T_H_2: T helper type 2 cell
TH17: T helper type 17 cell
TSLP: Thymic stromal lymphopoietin

## References

[1] Bousfiha A, et al. The 2022 update of IUIS Phenotypical classification for human inborn errors of immunity. J Clin Immunol 2022;42(7):1508–20. 36198931 10.1007/s10875-022-01352-z

[2] Petrova E, Hovnanian A. Advances in understanding of Netherton syndrome and therapeutic implications. Expert Opin Orphan Drugs 2020;8(11):455–87.

[3] Hovnanian A. Netherton syndrome: skin inflammation and allergy by loss of protease inhibition. Cell Tissue Res 2013;351(2):289–300. 23344365 10.1007/s00441-013-1558-1

[4] Zambruno G. Orphanet: Netherton syndrome 2008. [Cited 31 March 2024]. https://www.orpha.net/en/disease/detail/634

[5] Olbrich H, et al. Dupilumab in inflammatory skin diseases: a systematic review. Biomolecules 2023;13(4):634. 37189381 10.3390/biom13040634 PMC10136243

[6] Sun Q, et al. Development and initial validation of a Novel System to assess ichthyosis severity. JAMA Dermatol 2022;158(4):359–65. 35171201 10.1001/jamadermatol.2021.5917 PMC8851366

[7] Yan AC, et al. The safety and efficacy of pimecrolimus, 1%, cream for the treatment of Netherton syndrome: results from an exploratory study. Arch Dermatol 2010;146(1):57–62. 20083693 10.1001/archdermatol.2009.326

