# Supplementary material for: Dupilumab in a 9-week-old with Netherton Syndrome Leads to Deep Symptom Control
Source: J Clin Immunol. 2024 Nov 15;45(1):42. doi: 10.1007/s10875-024-01837-z (PMC11568019; doi:10.1007/s10875-024-01837-z)
Supplement: Supplementary file 1 — (DOCX. 12.4 KB) [file 10875_2024_1837_MOESM1_ESM.docx]

**Supplementary case report**

Here we describe the progress of the disease with complications up to treatment with dupilumab. Due to the high transdermal fluid loss and thermolability, the newborn had to be maintained in an incubator with 60 % moisture. Elevated inflammation values and the detection of *MRSA* in the skin swabs and blood culture necessitated intravenous antibiotic treatment. Insufficient weight gain and thin stools led to a change of nutrition to an extensively hydrolyzed formula. The patient's hair fell out in the 6th week of life. A falling hemoglobin level without signs of bleeding led to an erythrocyte transfusion. The skin was cleaned four times daily with NaCl 0.9% and chlorhexidine solution. Open wounds were treated with eosin 2% solution. In addition, full baths were performed twice daily with potassium permanganate. In the further course, skincare was changed to several daily creams with dexpanthenol and daily bathing with moisturizing oil, and once weekly bathing with octenidine.
